# Marfan Syndrome beyond Aortic Root—Phenotyping Using Cardiovascular Magnetic Resonance Imaging and Clinical Implications

**DOI:** 10.3390/medicina59050942

**Published:** 2023-05-14

**Authors:** Evangelia Nyktari, Maria Drakopoulou, Panagiotis Rozos, Sofia Loukopoulou, Thomas Vrachliotis, Soultana Kourtidou, Konstantinos Toutouzas

**Affiliations:** 1 Onassis Cardiac Surgery Center, 17674 Athens, Greece; 2 Cardiology Clinic, ‘Hippokration’ General Hospital, School of Medicine, National and Kapodistrian University of Athens, 11527 Athens, Greece; 3 Paediatric Cardiology Clinic, ‘Agia Sofia’ General Paediatric Hospital, 11527 Athina, Greece

**Keywords:** Marfan syndrome, arrhythmias, heart failure, cardiomyopathy, cardiovascular magnetic resonance imaging

## Abstract

Marfan syndrome (MFS) is an inherited autosomal-dominant connective tissue disorder with multiorgan involvement including musculoskeletal, respiratory, cardiovascular, ocular, and skin manifestations. Life expectancy in patients with MFS is primarily determined by the degree of cardiovascular involvement. Aortic disease is the major cardiovascular manifestation of MFS. However, non-aortic cardiac diseases, such as impaired myocardial function and arrhythmia, have been increasingly acknowledged as additional causes of morbidity and mortality. We present two cases demonstrating the phenotypical variation in patients with MFS and how CMR (Cardiovascular Magnetic Resonance) could serve as a “one stop shop” to retrieveS all the necessary information regarding aortic/vascular pathology as well as any potential underlying arrhythmogenic substrate or cardiomyopathic process.

## 1. Introduction

Marfan syndrome (MFS) is a relatively common (1 case in every 3–5000 people) autosomal-dominant genetic disorder, typically (90%) caused by mutations in the FBN1 gene encoding fibrillin 1 [1,2]. Fibrillin 1 is a large extracellular matrix glycoprotein that polymerizes into microfibrils. Fibrillin microfibrils contribute to the integrity and function of all connective tissues.

The syndrome involves musculoskeletal, cardiovascular, and ocular systems and is characterized by skeletal abnormalities, such as long bone overgrowth, dislocation of the ocular lens, pneumothorax, mitral valve disease, and aortic root dilatation. Due to its phenotypic variance, the diagnosis is based on the Ghent criteria [3], which are of immense importance especially in the case of limited access to genetic testing. In addition, the revised nosology puts more weight to cardiovascular complications, namely aortic root aneurysm, aortic dissection, and ectopia lentis, which represent cardinal features of the syndrome (Table 1) [4].

Aortic disease is the major cardiovascular manifestation in MFS with aortic dissection being the most common cause of death. However, non-aortic cardiac disease, most notably in the form of mitral valve prolapse, often complements the phenotype. Recently, impaired myocardial function and arrhythmias have drawn attention, as they have been increasingly reported as additional causes of morbidity and mortality in large MFS surveillance groups [5,6,7,8]. Cardiovascular imaging is therefore fundamental to the screening, diagnosis, and life-long follow-up of this population.

We describe two cases of young boys with Marfan syndrome and cardiovascular disease beyond aortic involvement. The role of Cardiovascular Magnetic Resonance Imaging (CMR) is highlighted not only at demonstrating aorta disease but also at assessing valvular and ventricular function and characterizing myocardial tissue and potential arrhythmogenic substrate (arrhythmogenic mitral valve prolapse, myocardial function, and ventricular arrhythmias).

## 2. Case 1—Aorta and Mitral Valve Prolapse

**Figure 1:**
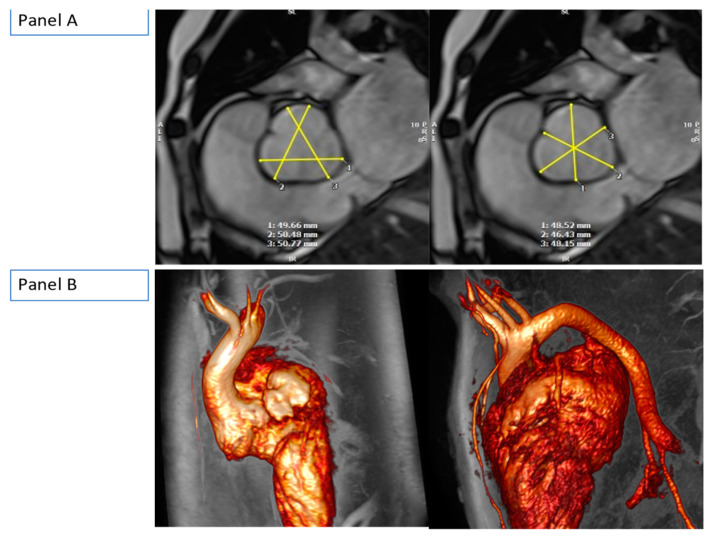
(**Panel A**): CMR balanced steady-state free precession (b-SSFP) measurement of the aortic root. Both sinus-to-sinus (**left-sided** image) and sinus-to-commissure (**right-sided** image) measurements are shown. (**Panel B**): CMR gadolinium-enhanced (GE) angiogram of the entire thoracic aorta.

A 17-year-old young boy with diagnosed Marfan syndrome at the age of 7, presented at the outpatient clinics with progressive exertional dyspnoea, fatigue, and severe functional status impairment. The diagnosis of Marfan syndrome has been based on clinical criteria (Ghent criteria) with no genetic testing. The physical exam was remarkable for severe kyphosis and scoliosis, severe pectus excavatum, systolic murmur, and arachnodactyly. Electrocardiogram (ECG) recordings were on normal sinus rhythm with frequent ventricular extra-systoles (RBBB morphology, superior axis (QS in leads II, III, aVF), and late transition at leads V4–V5). Transthoracic echocardiography showed severe aortic root dilatation (~53 mm) while the ascending aorta was of normal size (29 mm). The aortic valve was functioning well. There was severe mitral regurgitation (MR) due to bileaflet prolapse. The left ventricle (LV) was dilated with impaired systolic function (ejection fraction (EF)~44% by Simpsons’ biplane) in the presence of significant volume overload. Based on European Society of Cardiology (ESC) guidelines, the patient fulfilled criteria for aortic root and mitral valve surgery. The patient was referred for a pre-operative CMR scan to assess both the entire aorta as well as the severity of MR and LV function. CMR confirmed aortic root dilatation of 52 mm with a z-score of 6.4 (Figure 1, Video S1).

**Figure 2:**
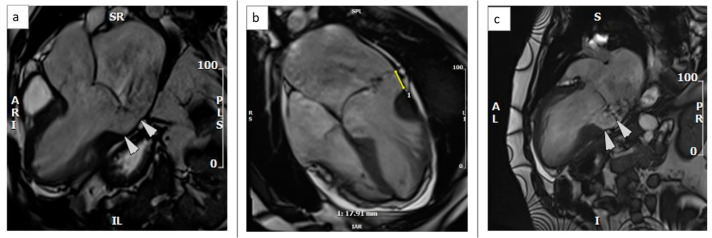
CMR balanced steady-state free precession (b-SSFP) cines in systole showing mitral annular disjunction (18 mm between arrows): (**a**). Left ventricular outflow tract (LVOT) view, (**b**). heart-long-axis (HLA) view, (**c**). ventricle long-axis (VLA) view.

There was bileaflet mitral valve prolapse (MVP) with redundant mitral valve leaflets and prominent mitral annular disjunction (MAD) was apparent with a maximal disjunction distance of 18.0 mm (Figure 2, Videos S2 and S3).

**Figure 3:**
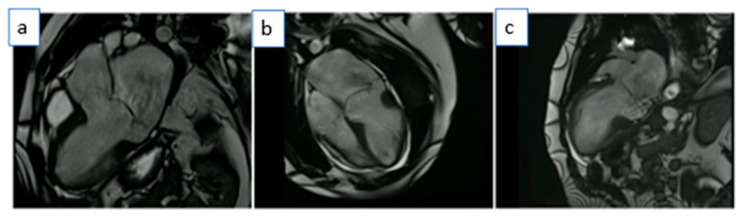
CMR b-SSFP still images in systole showing severe mitral valve regurgitation into a severely dilated left ventricle: (**a**). LVOT view, (**b**). HLA view, (**c**). VLA view.

Hyper-mobility of the basal inferolateral wall with hypokinesia of the adjacent posteromedial papillary muscle indicating increased mechanical tethering of the sub-valvular apparatus was noted. As a result, there was severe holosystolic mitral valve regurgitation (regurgitant fraction 62%) into a gigantic left atrium. The left ventricle was severely dilated with an ejection fraction (EF) of 53%. In the context of severe chronic volume overload, such an EF reflected at least mild–moderate systolic function impairment (Figure 3, Video S4).

**Figure 4:**
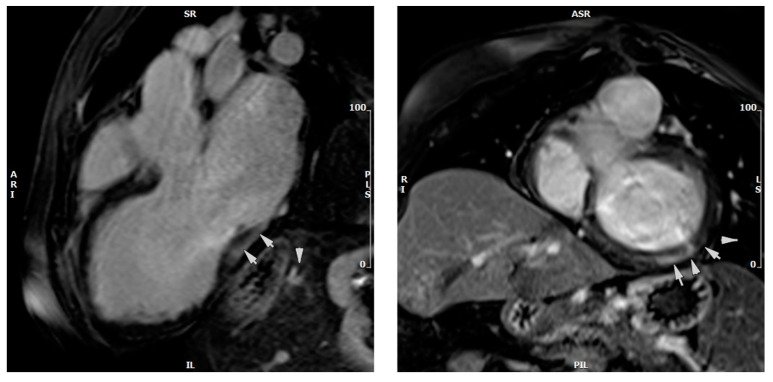
Late gadolinium images at LVOT view (**left-sided** image) and basal short-axis view (**right-sided** image) showing focal sub-epicardial/mid-wall enhancement (small arrows).

Late images post-gadolinium-injection (LGE) demonstrated focal sub-epicardial/mid-wall fibrosis (non-ischaemic pattern) at the basal inferolateral wall with involvement of the adjacent posteromedial papillary muscle (Figure 4) This pattern of fibrosis along with the electrical phenotype of ventricular extra-systoles (VEs) and the morphofunctional characteristics of MVP (MAD 18 mm, signs of increased tethering of sub-valvular apparatus) prompted the diagnosis of an arrhythmogenic phenotype. The patient was further evaluated with a 24 h Holter monitoring that showed frequent VEs (>5% VES burden) and one episode of Non-Sustained Ventricular Tachycardia (NSVT) involving five QRS complexes. The morphology of VES in ECG showed an RBBB configuration, superior axis (QS in leads II, III, aVF), and late transition at leads V4–V5.

**Figure 5:**
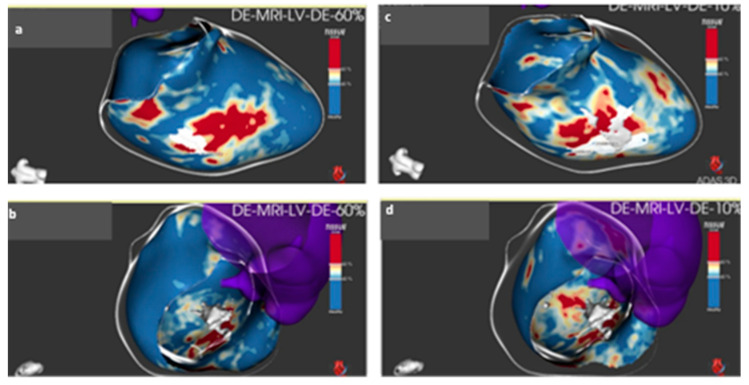
ADAS-LV scar segmentation analysis showing VT corridors: panels **a** and **b** showing a potential VT corridor at the layer 60% distance from the endocardial surface. Scarring is coded as red areas, healthy tissue as blue areas, and border zone as yellowish area. Panels **c** and **d** showing a VT channel at the base of posteromedial papillary muscle.

Three-dimensional (3D) high-resolution LGE-CMR images (ECG-gated free-breathing navigator, FLASH sequence, pixel size 1.5 mm × 1.5 mm × 2 mm, 1.5 T Sola Siemens Erlangen) were processed off-line using a dedicated software package (Automatic Detection of Arrhythmic Substrate, ADAS-VT, Galgo Medical SL, Barcelona, Spain) with semiautomatic determination of endo- and epicardial borders of the left ventricular myocardium. Subsequent automatic characterization of an internal 3D scar architecture was performed using a CMR-signal intensity distribution pattern. The LV myocardial wall was split into layers using 10% steps from the endocardium to the epicardium. Dense scar, heterogeneous tissue (“border zone”), and normal tissue were differentiated using default thresholds of >60% of the maximum pixel intensity, 40% to 60%, and <40%, respectively [9]. Within the fibrosis area, conducting channels were defined as a border zone corridor connecting normal tissue (“healthy-to-healthy”). ADAS-LV scar segmentation analysis was suggestive of a possible VT corridor in the basal inferolateral wall (60% layer from endocardium) and a second one at the base of the posteromedial papillary muscle (Figure 5, Video S5).

Overall, the patient had an arrhythmogenic mitral valve prolapse phenotype [8,10] with one high-risk feature from ECG recordings (NSVT) and three phenotypic risk features from imaging (MAD, redundant leaflets, severe left atrial dilatation, and fibrosis involving the mitral apparatus, i.e., posteromedial papillary muscle and adjacent basal inferolateral wall). The case was discussed in an MDT and the consensus was that an ICD could have been prophylactically implanted—after shared decision making with the patient. The patient was offered ICD implantation and surgery involving both aortic root and mitral valve replacement but unfortunately the patient refused any medical treatment and was lost to follow-up against any medical advice. Of note, a subcutaneous ICD would have been suitable for this patient as there was a low likelihood of needing pacing, and it might be less destructive to the tricuspid valve in the future, whilst securing safety. This can be considered, especially in centers experienced with subcutaneous ICD implantation.

## 3. Case 2—Heart Failure, Cardiomyopathy

A 16-year-old young boy, diagnosed with MFS as a neonate, under long-term therapy (from the age of 3) with beta-blockers and an ARB inhibitor due to aortic root dilatation (36 mm on echocardiography), was referred for a CMR scan due to a rapid deterioration of the LV function over the course of 1 year (LVEF drop from 60% to 20%, LVESD from 40 mm to 60 mm). An ECG showed a normal sinus rhythm with a pattern of Wolf Parkinson White (WPW) even though no arrhythmia was ever registered during 24 h Holter monitoring. The patient was in New York Heart Association (NYHA) functional class II.

**Figure 6:**
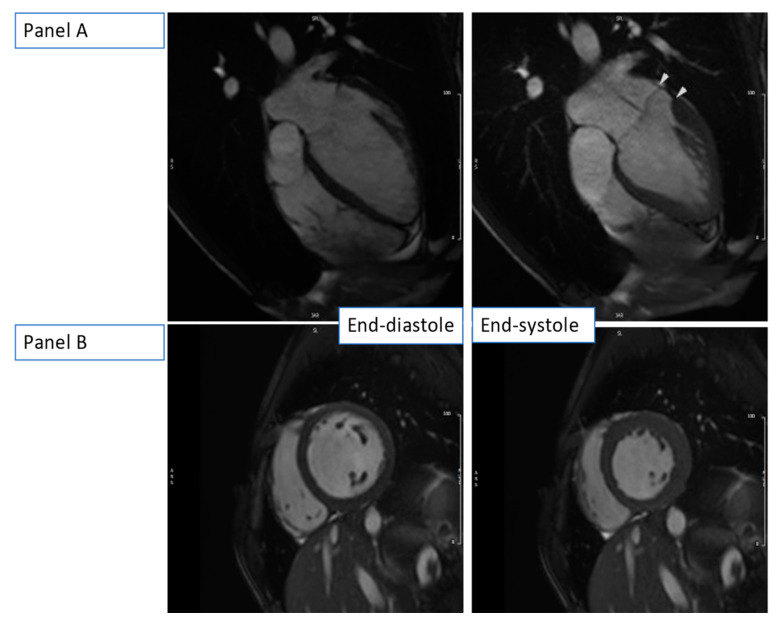
b-SSFP still images at end-diastole (**left-sided** images) and end-systole (**right-sided** images) showing dilated LV with severely reduced ejection fraction in the absence of volume load from valvular dysfunction. (**Panel A**): HLA view at end-diastole and end-systole with arrows showing MAD. (**Panel B**): Short-axis basal slice at end-diastole and end-systole.

CMR confirmed the dilation of the aortic root (36 mm, z-score of 3.65) (Figure 6, Video S6) and the presence of mitral valve prolapse with only mild end-systolic regurgitation (regurgitant fraction < 5%). Yet, there was significant MAD (approximately 11 mm) and signs of increased mechanical tethering of the sub-valvular apparatus (hypokinesia of the posteromedial papillary muscle). The LV was globular and mildly dilated with a severely reduced ejection fraction (LVEF 32%). Diffuse hypokinesia was noted, more prominent on the septum, which appeared to be thinned and almost akinetic. There was no valvular disease (no aortic regurgitation, only mild mitral valve regurgitation) to justify the severity of the LV dilatation and systolic function impairment.

**Figure 7:**
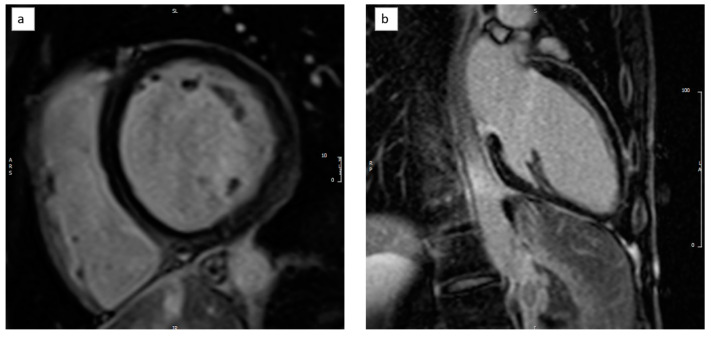
Late gadolinium images showing linear mid-wall LGE at the septum (and basal inferior/inferolateral walls (panels **a**,**b**).

Late gadolinium study (LGE) revealed prominent fibrosis of a non-ischaemic pattern involving both the thinned septum (linear mid-wall LGE) and the basal inferior and lateral walls (sub-epicardial/mid-wall LGE) (Figure 7). The fibrosis extent was calculated using the five standard deviation (SD) method, at approximately 11% of the total LV mass.

The diagnosis of underlying primary cardiomyopathy probably related to MFS was made and the patient was referred to the Advanced Heart Failure Team for medical treatment optimization and early pre-transplantation evaluation in case of no response to conservative management.

## 4. Discussion

Aortic root dilatation/dissection and mitral valve prolapse are established cardiovascular manifestations in MFS. However, heart failure and arrhythmia-related sudden cardiac death have recently emerged as additional causes of morbidity and mortality [7].

Our two cases demonstrate the phenotypical variation in patients with MFS and how CMR could serve as a “one stop shop” to retrieve all the necessary information regarding aortic/vascular pathology as well as any potential underlying arrhythmogenic substrate or cardiomyopathic process.

Cardiac imaging is of paramount significance for the diagnosis and long-term monitoring of the complex manifestations of MFS. The European guidelines (ESC) recommend using two modalities in each patient with echocardiography being the first-choice modality and either CMR or CT being the second [11]. Echocardiographic assessment of the aortic root includes measurements at the annulus, sinuses and sinotubular junction, distal ascending aorta, arch, and descending aorta. In adults, measurements are made at end-diastole using the leading-to-leading edge method and the values obtained are adjusted for sex, age, and body surface area. Apart from the detailed aortic assessment, valvular morphology (bicuspid aortic valve and presence of MVP) and ventricular dimensions and function must be examined, as well as the presence of a PDA. There is also a recommendation for CMR or CT angiography from head to pelvis in every patient at baseline covering the entire aorta and branches.

CMR is a radiation-free, multiplanar tomographic imaging technique that is not limited by acoustic windows (a common problem in the presence of severe musculoskeletal deformities) and can provide not only anatomical but also functional imaging of the heart and vessels as well as tissue characterization of the myocardium. These make it an ideal method for surveillance especially amongst the young and pregnant population avoiding repeated radiation exposure from serial CT scanning. It has been validated against echocardiography for aortic root measurement and its superiority at demonstrating the asymmetrical root dilatation often seen in MFS is well established [12]. With the use of ECG-gated non-contrast, respiratory, or self-navigated 3D imaging sequences, optimal delineation of the aortic wall [13] covering the entire length of aorta can be successful allowing for measurements at end-diastole in the same way as in echocardiography. For evaluation of the aortic root, the use of ECG-gated 2D cine b-SSFP imaging is preferred [14]. The measurement of the sinuses of Valsalva planes is planned from both the oblique sagittal and oblique coronal left ventricular outflow tract (LVOT) and axial planes. Axial planes should be perpendicular to the true axis of the aorta to avoid hyper-estimation. A contiguous stack of b-SSFP cine images, 5 mm in thickness with no gaps, covering the entire aortic root, is obtained. Sinus-to-sinus and sinus-to-commissure measurements taken at end-diastole are most comparable to corresponding echocardiographic measurements and reference values are available [15]. Gadolinium-enhanced (GE) aortography is not ECG-gated and unfortunately without ECG gating the root measurements are the least reliable so it should be avoided if possible for assessment of the aortic root. However, GE aortography is more sensitive for demonstrating dissection flaps and as a result two aortography data sets (one with and one without contrast) should be taken should no contraindication for gadolinium exist.

Furthermore, phase-contrast (PC) CMR provides a tool to non-invasively quantify blood flow and thus assess valvular function [16]. Cardiac valves, especially the aortic and mitral valves, are visualized by dynamic b-SSFP imaging in order to identify the morphology and function. The assessment and quantification of any stenosis or regurgitation is made by PC imaging through the valve or vessel of interest (through-plane velocity flow mapping). Through-plane velocity flow mapping at the STJ should be performed at the sinotubular junction to quantitatively assess aortic regurgitation or at the level of the aortic valve to assess for stenosis. In the presence of mitral valve prolapse, mitral valve regurgitation may be quantified by detracting the aortic forward flow (at STJ junction) from the LV stroke volume (mitral regurgitant volume). More recently, time-resolved flow imaging with velocity encoding along all three flow directions and 3D anatomic coverage (4-Dimensional flow MRI) has enabled comprehensive 3D visualization and quantification of haemodynamics throughout the circulatory system, including the semilunar or atrioventricular valves.

CMR is considered the gold-standard technique for biventricular anatomy and function assessment and can detect any mild “DCM-like” phenotypes of MFS cardiomyopathy. Early imaging studies have provided data supporting the presence of a primary myocardial impairment in the absence of valvular disease or cardiovascular surgery in patients with MFS [5]. Tissue characterization with the use of gadolinium can provide information of underlying fibrosis or scarring that could potentially serve as arrhythmogenic substrate. Furthermore, recent developments in CMR post-processing (ADAS-VT, Galgo Medical SL, Barcelona, Spain) allows for detailed 3D scar characterization including size (scar/fibrosis burden), transmurality (endocardium vs. epicardium), and composition (scar core vs. border zone) [17]. Thresholding-based analysis of high-resolution 3D-LGE images is used to create LV models consisting of concentric layers from endocardium to epicardium that are color-coded according to pixel signal intensity (PSI) using a full-width-at-half-maximum algorithm. The hyper-enhanced myocardium on the PSI maps is sub-characterized using a 40–60% configuration where <40% is healthy tissue, 40–60% is border zone (BZ), and >60% is scar core. VT corridors are identified as regions of the BZ surrounded by scar core [18]. These VT corridors are the structural equivalent of the electrical conduction channels identified by voltage mapping and represent the VT isthmus corridors. The produced ADAS-based maps from the LGE images can subsequently be merged with the electroanatomical maps created at the electrophysiology lab and help to guide VT ablation procedures.

In the first case, the fully expressed Marfan phenotype was well captured by CMR imaging, depicting the need for aortic and mitral valve surgery as well as taking measures to protect the patient from arrhythmic sudden cardiac death. Aortic surgery is indicated in patients with MFS when the aortic sinus diameter is equal to or greater than 50 mm (Class I, Level C) and should be considered in those with an aortic root diameter equal to or greater than 45 mm and additional risk factors (family history of aortic dissection at a low diameter or personal history of spontaneous vascular dissection, progressive AR, desire for pregnancy, uncontrolled hypertension, and/or aortic size increase > 3 mm on repeated measurements using the same ECG-gated technique) or with a TGFBR1 or 2 mutation (Class IIa, Level C) [11].

Mitral valve surgery is indicated (Class I, Level B) either in symptomatic patients with severe chronic MR or in asymptomatic patients with LV dysfunction (LV end-systolic dimension (ESD) ≥ 40 mm and/or LVEF ≤ 60%) as in our first patient. Surgery should also be considered in asymptomatic patients with preserved LV function (LVESD < 40 mm and LVEF > 60%) and atrial fibrillation secondary to mitral regurgitation or pulmonary hypertension, or if the surgical risk is low and there is significant LA dilatation (Class IIa, Level B).

Mitral valve prolapse in Marfan is linked with age, with up to 75% affected beyond the age of 60 years. In the setting of MVP, the presence of significant MAD (>7 mm), signs of increased tethering, and fibrosis of the inferior/inferolateral wall and adjacent posteromedial papillary muscle have been described as part of an arrhythmogenic MVP sub-type [10]. The arrhythmic mitral valve complex is defined by the presence of MVP (with or without MAD), combined with frequent and/or complex VA in the absence of any other well-defined arrhythmic substrate (such as active ischaemia, underlying cardiomyopathy, or scarring due to other defined pathology). It seems that MAD is an important component of this phenotype, especially in Barlow mitral valves, linked to more ventricular arrhythmias, whereas the arrhythmic outcome in this sub-population has been shown to be independent of age, MR severity, bileaflet prolapse, and LVEF [19].

Of note, in syndromic MVP, MAD prevalence has been reported to be up to 34% for MFS especially in patients with documented ventricular arrhythmias [20]. Analysis of 3D scar architecture and conducting channels (corridors) by high-resolution LGE imaging provides further support for the presence, extent, and exact location of arrhythmogenic substrate in this high-risk population. In our first patient, the morphology of VES (RBBB morphology, superior axis (QS in leads II, III, aVF), and late transition at leads V4–V5) is in agreement with the VT corridor at the base of the posteromedial papillary muscle. This could represent a target for VT ablation.

In the second case, CMR imaging unveiled the underlying cardiomyopathic process probably related to the MFS and provided a robust quantification of the extent of replacement fibrosis (non-reversible) thus ensuring timely referral to the Advanced Heart Failure team. LV dimensions and geometry are normal in most MFS patients. However, up to 7% have LV dilatation without other features of idiopathic dilated cardiomyopathy [12]. Furthermore, there is a growing body of evidence that up to 8% of all MFS cases will develop secondary LV dysfunction due to significant valvular dysfunction (aortic or mitral valve regurgitation) or ischaemic heart disease, while primary LV dysfunction can be found in 3% of the MFS population [21,22,23].

## 5. Conclusions

The presence of non-aortic cardiovascular disease in patients with MFS must be in focus in the caretaking of patients. Arrythmia risk stratification in patients with MSF can be challenging, emphasizing the need for more research in this area.

Efforts have been made to identify risk markers of malignant arrhythmias and underlying cardiomyopathy in this population. CMR should be an integral part of the imaging in MFS, as it covers a broad spectrum of cardiovascular complications and dictates the timing and nature of intervening.

## Figures and Tables

**Table 1:** Simplified Ghent criteria for MFS diagnosis.

Family History of MFS Absent	Family History of MFS Present
Aorta (Z ≥ 2 or dissection) and ectopia lentis	Ectopia lentis
Aorta (Z ≥ 2 or dissection) and a causal FBN1 mutation	Systemic score ≥ 7
Aorta (Z ≥ 2 or dissection) and systemic features (≥7)	Aorta (Z ≥ 2 above 20-year-old, Z ≥ 3 below 20 year, or dissection)
Aorta (Z ≥ 2 or dissection) and systemic features (≥7)	

Z-score is derived from a measurement of the sinus of Valsalva indexed to body surface area. Caveat: other conditions such as Loeys–Dietz syndrome must be excluded.

## Data Availability

Not applicable.

## Supplementary Materials

The following are available online at https://www.mdpi.com/article/10.3390/medicina59050942/s1, Video S1 Case 1: CMR b-SSFP axial cine of the aortic root; Video S2 Case 1: CMR b-SSFP LVOT view showing aortic and mitral regurgitation; Video S3 Case 1: CMR b-SSFP HLA (Heart Long axis) view showing mitral valve prolapse, mitral annular disjunction and mitral regurgitation; Video S4 Case 1: CMR b -SSFP VLA (Ventricle Long axis) view showing mitral valve prolapse, mitral annular disjunction and mitral regurgitation; Video S5 Case 1: ADAS-LV scar segmentation analysis showing a VT-corridor in the basal inferolateral wall (60% layer from endocardium), and a second one at the base of the posteromedial papillary muscle; Video S6 Case 2: CMR b-SSFP LVOT view.
