# Supplementary figures and images for: Marfan Syndrome beyond Aortic Root—Phenotyping Using Cardiovascular Magnetic Resonance Imaging and Clinical Implications
Source: Medicina (Kaunas). 2023 May 14;59(5):942. doi: 10.3390/medicina59050942 (PMC10223153; doi:10.3390/medicina59050942)

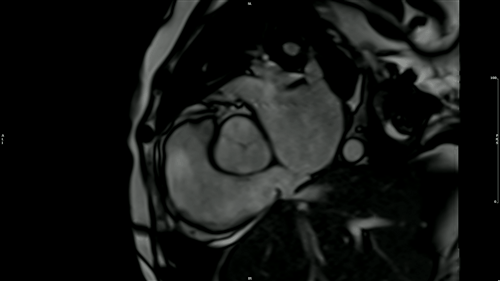

Supplement: Supplementary file 1 [file medicina-59-00942-s001.zip › Video S1.gif]

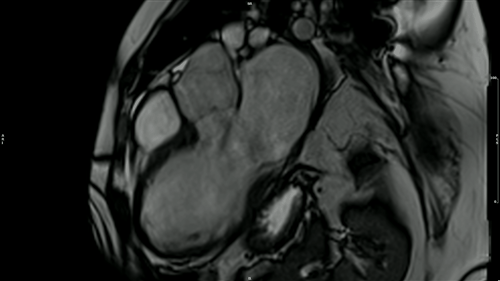

Supplement: Supplementary file 1 [file medicina-59-00942-s001.zip › Video S2.gif]

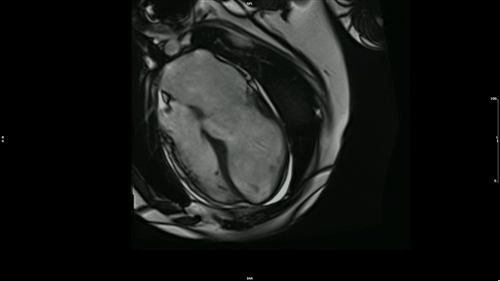

Supplement: Supplementary file 1 [file medicina-59-00942-s001.zip › Video S3.gif]

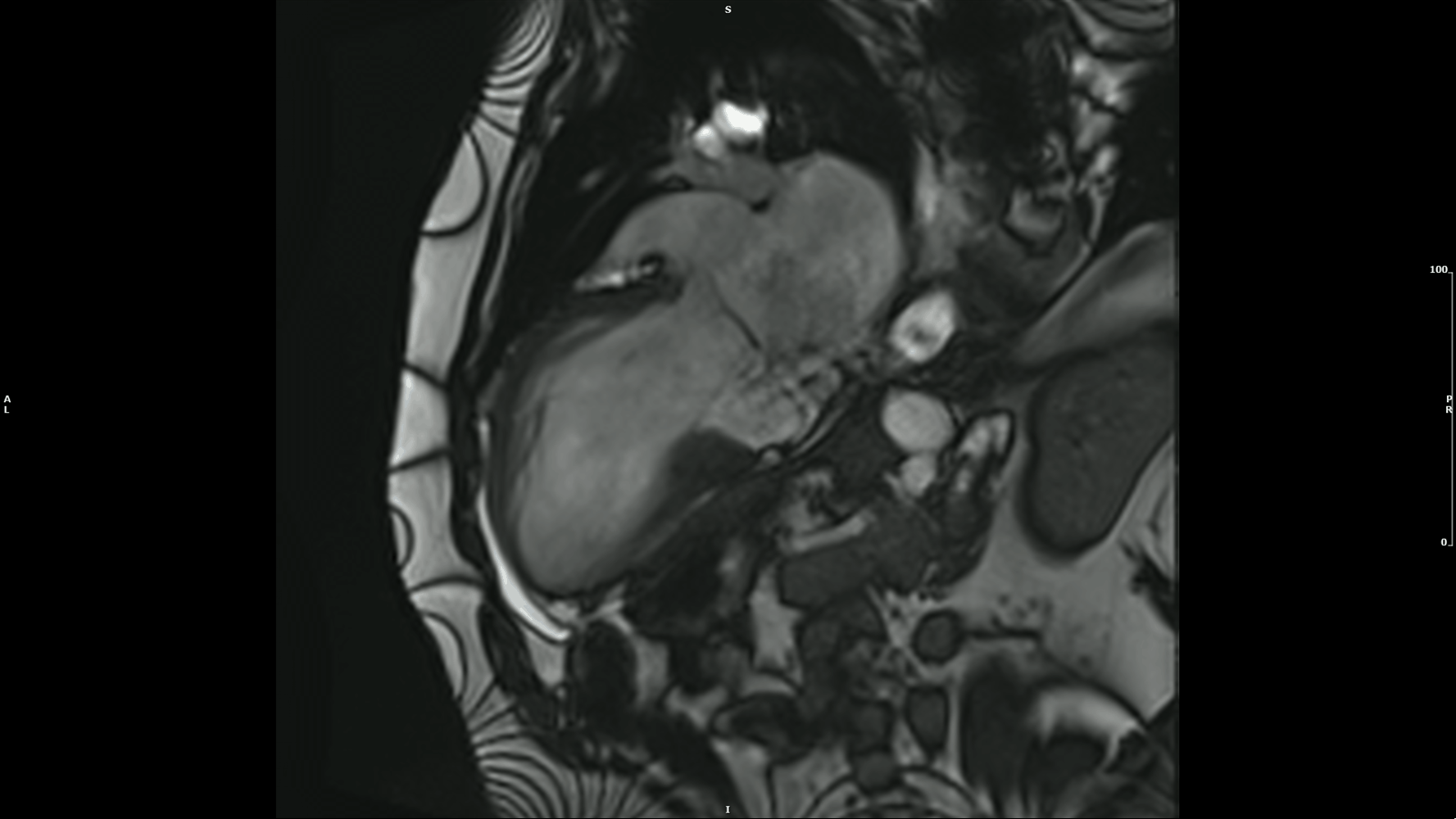

Supplement: Supplementary file 1 [file medicina-59-00942-s001.zip › Video S4.gif]

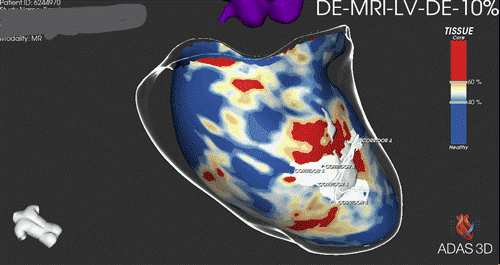

Supplement: Supplementary file 1 [file medicina-59-00942-s001.zip › Video S5.gif]

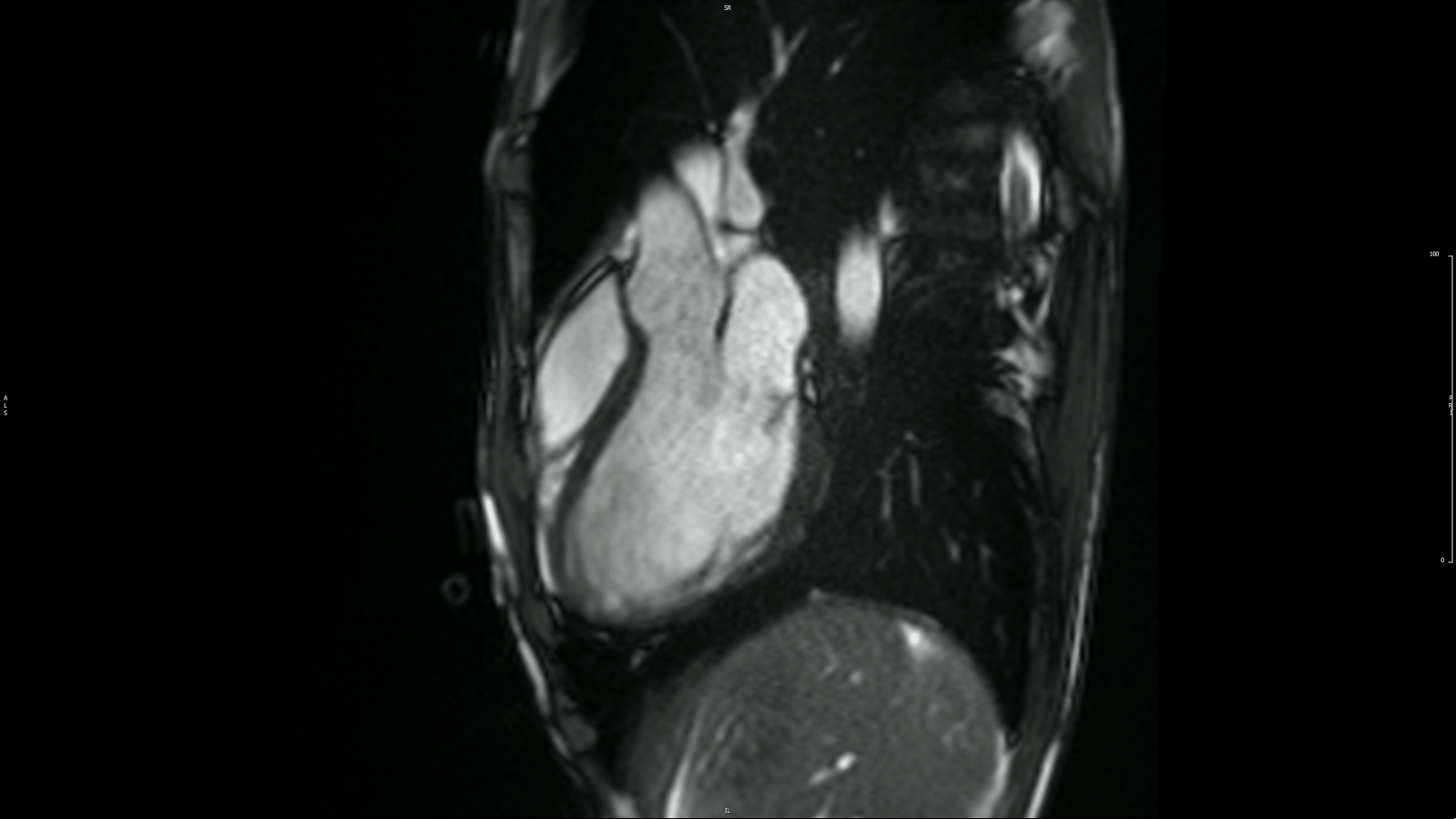

Supplement: Supplementary file 1 [file medicina-59-00942-s001.zip › Video S6.gif]
